# A Reference-Free Method for Brightness Compensation and Contrast Enhancement of Micrographs of Serial Sections

**DOI:** 10.1371/journal.pone.0127855

**Published:** 2015-05-28

**Authors:** Shi-Jie Chang, Shuo Li, Arne Andreasen, Xian-Zheng Sha, Xiao-Yue Zhai

**Affiliations:** 1 Department of Biomedical Engineering, China Medical University, Shenyang, Liaoning, China; 2 Department of Biochemistry, China Medical University, Shenyang, Liaoning, China; 3 Department of Biomedicine—Anatomy, Aarhus University, Aarhus, Denmark; 4 Department of Histology and Embryology, China Medical University, Shenyang, Liaoning, China; Glasgow University, UNITED KINGDOM

## Abstract

Three-dimensional (3D) reconstruction of an organ or tissue from a stack of histologic serial sections provides valuable morphological information. The procedure includes section preparation of the organ or tissue, micrographs acquisition, image registration, 3D reconstruction, and visualization. However, the brightness and contrast through the image stack may not be consistent due to imperfections in the staining procedure, which may cause difficulties in micro-structure identification using virtual sections, region segmentation, automatic target tracing, etc. In the present study, a reference-free method, Sequential Histogram Fitting Algorithm (SHFA), is therefore developed for adjusting the severe and irregular variance of brightness and contrast within the image stack. To apply the SHFA, the gray value histograms of individual images are first calculated over the entire image stack and a set of landmark gray values are chosen. Then the histograms are transformed so that there are no abrupt changes in progressing through the stack. Finally, the pixel gray values of the original images are transformed into the desired ones based on the relationship between the original and the transformed histograms. The SHFA is tested on an image stacks from mouse kidney sections stained with toluidine blue, and captured by a slide scanner. As results, the images through the entire stack reveal homogenous brightness and consistent contrast. In addition, subtle color differences in the tissue are well preserved so that the morphological details can be recognized, even in virtual sections. In conclusion, compared with the existing histogram-based methods, the present study provides a practical method suitable for compensating brightness, and improving contrast of images derived from a large number of serial sections of biological organ.

## Introduction

Three-dimensional (3D) reconstruction of organs and tissues based on serial sections is a well-established. Compared with traditional morphological approaches, its advantage is that computer assisted 3D reconstructions can visualize micro-structures in space, provide abundant morphological information, and localize accurately cellular functional proteins. This technique has been successfully used in kidneys [1–3], nerves [4], limbus [5], etc.

In order to apply this technique, high quality of micrographs is needed, i.e. homogeneity of brightness, contrast, and resolution through the entire image stack derived from a large number of biological serial sections must be ensured. The gray value variance of two corresponding pixels in the neighboring images will result in artificial stripes in computer-aided *resliced* images (virtual section rendered from the image stack), disturbing the accurate region segmentation and automatic target tracking of the structure. This may ultimately cause distortions of the reconstructed structures. Such defects are inevitable, especially in images from sections stained in different batches and different photographing conditions.

Currently, most algorithms for correcting the variance of contrast and brightness of serial images are designed for images from confocal laser scanning microscope (CLSM), CT, or MRI. In the case of CLSM. When CLSM images from a thick specimen are captured, their brightness may decline with the depth of the specimen because of signal attenuation due to scattering, absorption, or photo bleaching [6, 7]. This weakness could be overcome by finding functions of brightness, e.g. intensity decay function [8] and point spread function [9]. In the case of CT or MRI, the brightness intensity may also be inconsistent through the image stack, even if the same patient is examined in the same scanner and with the same protocol. This may cause artifacts and errors in acquiring quantitative information [10,11], image registration [12], and region segmentation. Nyúl et al. have proposed [13] and later improved [14] a histogram specification algorithm according to the standard histogram derived from different MRI image stacks. The algorithm consists of two steps: a training step and a transform step. In the first step, a stack of images obtained from the same body region and protocol corresponding to a population of patients is given as input. Then the parameters (landmarks) of a ‘‘standard” reference histogram are calculated. In the second step, any given set of images acquired according to the protocol and for the body region can be corrected utilizing the information of the reference histogram, which is so called histogram specification. For a stack of CLSM images, Stanciu et al. [15] have designed an estimator ARDE (Automated Reference Detection Estimator) for optimizing the steps for selection of a reference image, and for creating a reference histogram. The ARDE contains three vectors describing the brightness, standard deviation, and mean value of the pixel grey values of the Sobel-operator-processed image.

The algorithms mentioned above are often integrated as software packages into the operating system of the instruments. However, the methods for CLSM, CT, and MRI are not suitable for a large number of images with severe and irregular variance in brightness and contrast, captured from biomedical serial sections that have been stained manually. In other words, a practical reference model (functions or reference images) can hardly be established for such image series, since the brightness and contrast vary irregularly from image to image, due to the section staining, i.e. no “*apriori*” image can be created or found. Thus, a self-adaptive algorithm dealing with the gray values is needed in order to handle images originating from serial sections.

In the present study, we propose an efficient reference-free method for automatic correction of irregular brightness and contrast deviation in serial images of microscopic sections. The processed images can be better aligned, and segmented precisely for further structural tracing and 3D reconstruction, as well as morphological qualification and quantification analysis.

## Materials and Methods

### Tissue Preparation and Image Acquisition

In the present study, all the images were obtained from developing mouse kidneys. The animal experiments were performed in accordance with the code of Ethics of World Medical Association (Declaration of Helsinki) and were approved by the Medical Ethics Committee of China Medical University.

C57/BL/6J mouse kidneys from three embryonic 17^th^ day fetuses (E17) and from three postnatal 7^th^ day pups (P7) were studied. The fetal kidneys were removed a few minutes after injection of pentobarbital sodium (50 mg/kg body weight) into the peritoneal cavity of the pregnant mice. The pup kidneys were preserved by perfusion fixation through the heart with a mixture solution of 4% paraformaldehyde and 1% glutaraldehyde in 0.06 M sodium cacodylate buffer, pH 7.4. All the kidneys or tissue blocks were further fixed for 1 h in 1% OsO_4_ in 0.1 M sodium cacodylate buffer, and embedded in Epon 812, then cut into serial semi-thin (2.5-μm-thick) sections using a Reichert Ultracut microtome (Reichert, Wienna, Austria). These consecutive sections were stained with toluidine blue for morphological recognition. In all, about 600 sections from each of the three E17 kidneys and about 800 sections from each of the P7 kidneys were obtained. The serial sections were used for the purpose of the 3D reconstruction and functional analysis of the mouse developing kidneys, as previously reported [16].

Stacks of the tissue images were captured using a slide scanner (NanoZoomer Digital Pathology, Hamamatsu, Japan) equipped with a 40× objective lens. The original image size was 10240 × 8196 pixels, whereby each pixel corresponded to 0.46 μm × 0.46 μm.

### Sequential Histogram Fitting Algorithm

**Fig 1:**
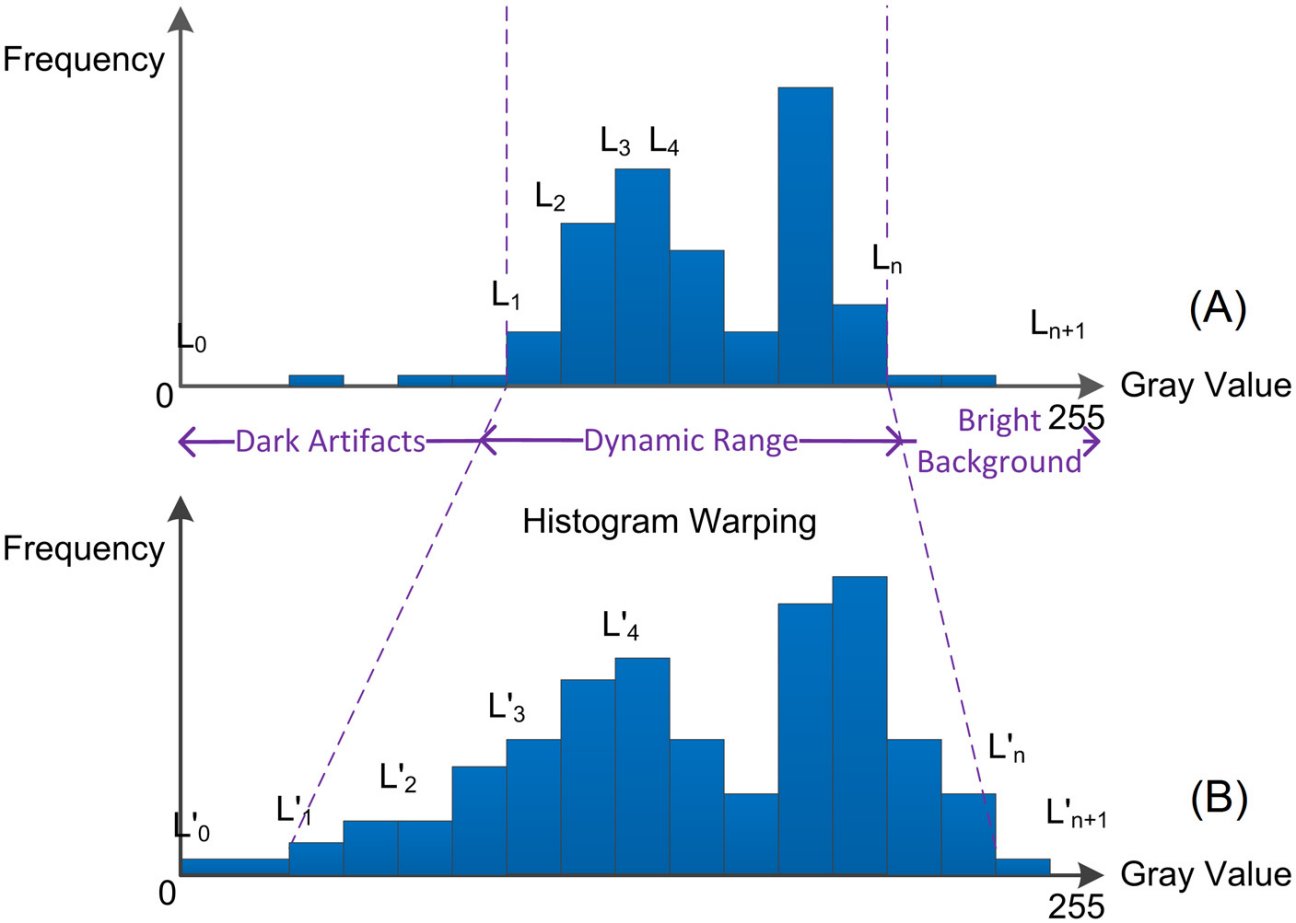
Schematic diagram of SHFA. (A) The histogram of the original image; (B) The desired histogram. The desired image is generated by gray value transformation from the original histogram to the desired histogram.

In the brightness histogram of a microscopic image (Fig 1), the columns on the leftmost part represent the frequencies of the pixels with the lowest gray values, which normally corresponds to the artifacts in the image; while those on the rightmost part represent the frequencies of the pixels with the highest gray values, corresponding to the bright background without tissue in the image; the middle part of the histogram represents the dynamic range, reflecting the frequencies of the gray values of the tissue structures. So, the dynamic range varies in shape from image to image. For example, the renal tubule wall is stained dark, which is reflected on the left part of the dynamic range; while the lumen of the tubules in kidney tissue is either not stained or stained very light, which is reflected on the right part of the dynamic range.

Most algorithms for adjustment of brightness and contrast are based on histogram operations, e.g. the shifting operation for brightness compensation, and the extension operation for contrast enhancement. However, with respect to a series of images, one single operation, either shifting or extending, may not meet the demands for both brightness and contrast homogenization at the same time. Therefore, the strategy is to take both brightness and contrast adjustment into consideration at the same time.

The principle of our method is that the shape of each histogram is only related to the morphological tissue structures in the image; so under optimal conditions of tissue preparation and image acquisition, the individual histograms should be consecutive and vary gradually between neighboring images. Therefore, a series of ideal histograms representing the consecutive images could be calculated.

***Sequential Histogram Fitting Algorithm*** (SHFA) was designed to guide the gray value transformation from the original histograms into the desired histograms according to the fitted variance features in the form of landmarks. So, when the series of original images were subjected to the series of the desired histograms, a series of desired images were obtained, in which the brightness and contrast were adjusted. So, the processed images may better reflect the real tissue morphology than the raw captured images in visualization and future analysis.

We denoted the images in the original and the desired stacks as I_*i*_ and I'_*i*_, where, *i* represents the image number in the original and desired image stacks. The histogram of I_*i*_ and I'_*i*_ were denoted by H_*i*_ and H'_*i*_. In order to preserve the basic shape of a histogram when transforming from the original histogram H_*i*_ into the desired histogram H'_*i*_, landmarks L_*i j*_ and L'_*i j*_ (recorded as the gray value where the landmark was set up in the histogram) were necessarily set up in the histograms H_*i*_ and H'_*i*_, where *j* represents the number of landmark from left to right in each histogram.

The main procedures for obtaining the series of desired images are to be described in two steps: (1) Histogram Fitting and (2) Gray Value Transformation.

Step 1. Histogram Fitting

 The overall algorithm of the step of “*Histogram Fitting*” is briefly described below:

**Algorithm for Step 1: Histogram Fitting**

**Input:** Original stack of images I_*i*_ (*i* = 1, 2, …, N);

**Output:** Landmarks in original histograms L_*i j*_ and landmarks in desired histograms L'_*i j*_ (*i* = 1, 2, …N, *j* = 0,1, 2, …, M+1);

**Begin:**

1.  **for** *i* = 1 to N **do**

2.   compute the histogram H_*i*_ of I_*i*_

3.   set up Landmarks L_*i j*_ (*j* = 0,1, 2, …, M+1)

4.  **endfor**

5.  **for** *j* = 0 to M+1 **do**

6.   compute the *j*th fitting curve based on L_*i j*_ (i = 1, 2, …, N) using fitting algorithm

7.   get the desired landmarks L'_*i j*_ from the computed fitting expression

8.  **endfor**

Firstly, all the histograms of the images in the original stack were calculated, and stored according to their image number. In order to gain both speed and accuracy, thumbnail images were processed instead of the original-sized images in this step.

Next, landmarks in each histogram were set up in order to preserve its basic shape. For landmarks L_*i j*_ in the original histogram H_*i*_, the leftmost point of the whole histogram, representing the lowest gray value (usually 0), was defined as L_*i*0_, and the rightmost point representing the highest gray value (usually 255) was defined as L_*i m+*1_. The leftmost point of the dynamic range (containing useful tissue information) was defined as L_*i* 1_, which was selected by searching the starting point where the gray value frequency steadily appeared. The rightmost point of the dynamic range was defined as L_*i m*_, which was selected by detecting the gray value of bright background.

The landmarks L_*i*2_, L_*i*3_, …, L_*i m*-1_ were set up by dividing the span between L_*i*1_ and L_*i m*_ in its corresponding cumulative histogram rather than the original histogram. Here, the cumulative histogram was transformed by integrating the original histogram, in which, the cumulative frequency *T*(*r*) were calculated as

$$T\left(r\right)=\int_{0}^{r}p_{r}\left(x\right)\text{d}x$$

where, *r* represents any gray value, and *p*_*r*_(*r*) represents the frequency of the gray value *r* in the original histogram. The integral of *p*_*r*_(*r*) from 0 to *r* was described as *T*(*r*) (0≤*r*≤255, 0≤*p*_*r*_(*r*)≤1, 0≤*T*(*r*)≤1).

The range from L_*i* 1_ to L_*i m*_ was divided evenly into *m*-1 spans according to the cumulative frequencies in the corresponding cumulative histogram. Then, the gray values corresponding to the landmarks of L_*i* 2_, L_*i* 3_, …, L_*i m*-1_ were determined. Finally, the basic shape of the original histogram H_*i*_ was delineated by all the landmarks {L_*i*0_, L_*i*1_, L_*i*2_, …, L_*i m*+1_}. The more landmarks, the more accurately the shape of the histogram was restored. Although there was a great variance of the landmark gray values in the original histogram H_*i*_, the proportions of the spans composed of the landmarks {L_*i* 0_, L_*i* 1_, L_*i* 2_, …, L_*i m*+1_} remained almost identical between the neighboring images, since the gray values were almost the same in the adjacent bio-tissue structures. So, the neighboring landmarks should be consecutive.

Thirdly, a fitting algorithm was used on the original landmarks in order to calculate the gray values of the desired landmarks in the serial of the histograms. The landmarks were grouped according to the landmark ordinate number *j*, the gray values of landmarks {L_1 *j*_, L_2 *j*_, …, L_*n j*_} could be fitted into a smoothed curve C_*j*_. Therefore, C_*j*_ was the gray value expression describing the gray values of desired landmarks {L'_1 *j*_, L'_2 *j*_, …, L'_*n j*_} as below:

$${\text{Gray}\;\text{value}\;\text{of}\;\text{L'}}_{i\;j}{\text{=}\;\text{C}}_{j}\left(i\right)\;\left(i=\;1,\;2,\;\ldots ,\;\text{N}\right)$$

Several complex and automatic curve fitting algorithms have been described [17,18]. When analyzing the bio-tissue images, cubic or quartic polynomial fittings[19] has proven to balance speed and accuracy of brightness compensation and contrast enhancement.

Finally, all the desired landmarks {L'_1 *j*_, L'_2 *j*_, …, L'_*n j*_} were calculated.

Step 2. Gray Value Transformation

 The overall algorithm of the step of “*Gray Value Transformation*” is briefly described below:

**Algorithm for Step 2: Gray Value Transformation**

**Input:** Original stack of images I_*i*_ (*i* = 1, 2, …, N); landmarks in the original and desired histograms L_*i j*_ and L'_*i j*_

**Output:** Desired stack of images I'_*i*_ (*i* = 1, 2, …, N)

**Begin:**

1.  **for** *i* = 1 to N **do**

2.   compute the desired histogram H'_*i*_ of I'_*i*_ according to H_*i*_ and the relationship between L'_*i j*_ and L_*i j*_

3.   transform the gray values of the original image I_*i*_ according to the relationship between H'_*i*_ and H_*i*_

4.  **endfor**

For the *i*th image, the desired landmarks {L'_*i* 0_, L'_*i* 1_, L'_*i* 2_, …, L'_*i m*+1_} and the original landmarks {L_*i* 0_, L_*i* 1_, L_*i* 2_, …, L_*i m*+1_} served as input. Now the desired histogram H'_*i*_ could be warped from the original histogram H_*i*_. A piecewise linear transformation was used in histogram warping, *i*.*e*., the gray values [L_*i j*_, L_*i j*+1_] in the original histogram were converted linearly into the gray values [L'_*i j*_, L'_*i j*+1_] in the desired histogram. However, the spans outside of the dynamic range, *i*.*e*. [L_*i* 0_, L_*i* 1_] and [L_*i* m_, L_*i* m+1_], might be compressed after warping. So, the gray value levels were compressed, especially the gray values near L_*i* 1_ and L_*i* m_, which might contain the useful dark or high-light information. To avoid this situation, the scale factors in the spans of [L_*i* 1_, L_*i* 2_] and [L_*i* m-1_, L_*i* m_] were adopted as the scale factors in the spans of [L_*i* 0_, L_*i* 1_] and [L_*i* m_, L_*i* m+1_] for linear transformation. The gray values below 0 or above 255 were ignored after warping. The operation above can be expressed as below:

Let piecewise scale factor be s_*i j*_ = (L'_*i j*+1_-L'_*i j*_) /(L_*i j*+1_-L_*i j*_); and the gray values in the original and desired histogram *i* be *x* and *x*':

$$x^{\prime }=\{\begin{array}{c} L_{i0}+s_{i1}\left(x-L_{i0}^{\prime }\right),if,x<L_{1}\\ L_{ij}+s_{ij}\left(x-L_{ij}^{\prime }\right),if,L_{1}\leq x\leq L_{m}\\ L_{im}+s_{im-1}\left(x-L_{im}^{\prime }\right),if,x>L_{m} \end{array}$$

Then,

$$x^{\prime }=\{\begin{array}{c} 0,if,x^{\prime }<0\\ x^{\prime },if,0\leq x^{\prime }\leq 255\\ 255,if,x^{\prime }>255 \end{array}$$

Now, any desired histogram H'_*i*_ was established. Finally, the desired image was generated by gray value transformation according to the relationship between H'_*i*_ and H_*i*_.

## Results

The algorithm above was implemented in C# (Visual Studio 2010 Express) and ran on a PC (Intel Core i7 720QM, 8GB). The algorithm was tested using an image stack obtained from either an E17 kidney or a P7 kidney.

### Sample 1: Image Stack of E17 Kidney

The image stack of E17 kidney was used to demonstrate the performance of the proposed algorithm.

**Fig 2:**
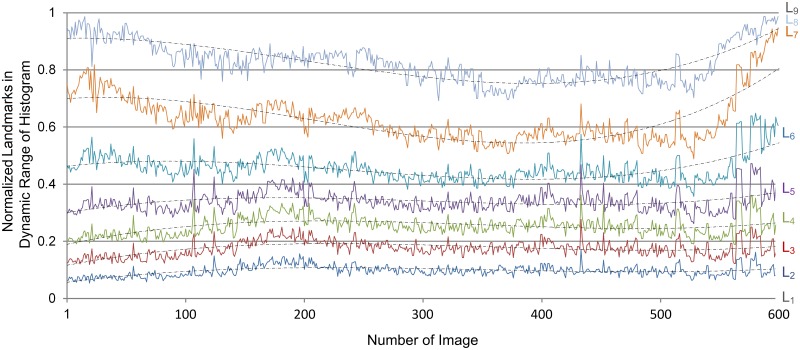
The normalized landmarks in the dynamic range of histogram. The solid lines represent the original landmarks and the dashed lines represent the fitted landmarks in histograms of the image stack.

**Fig 3:**
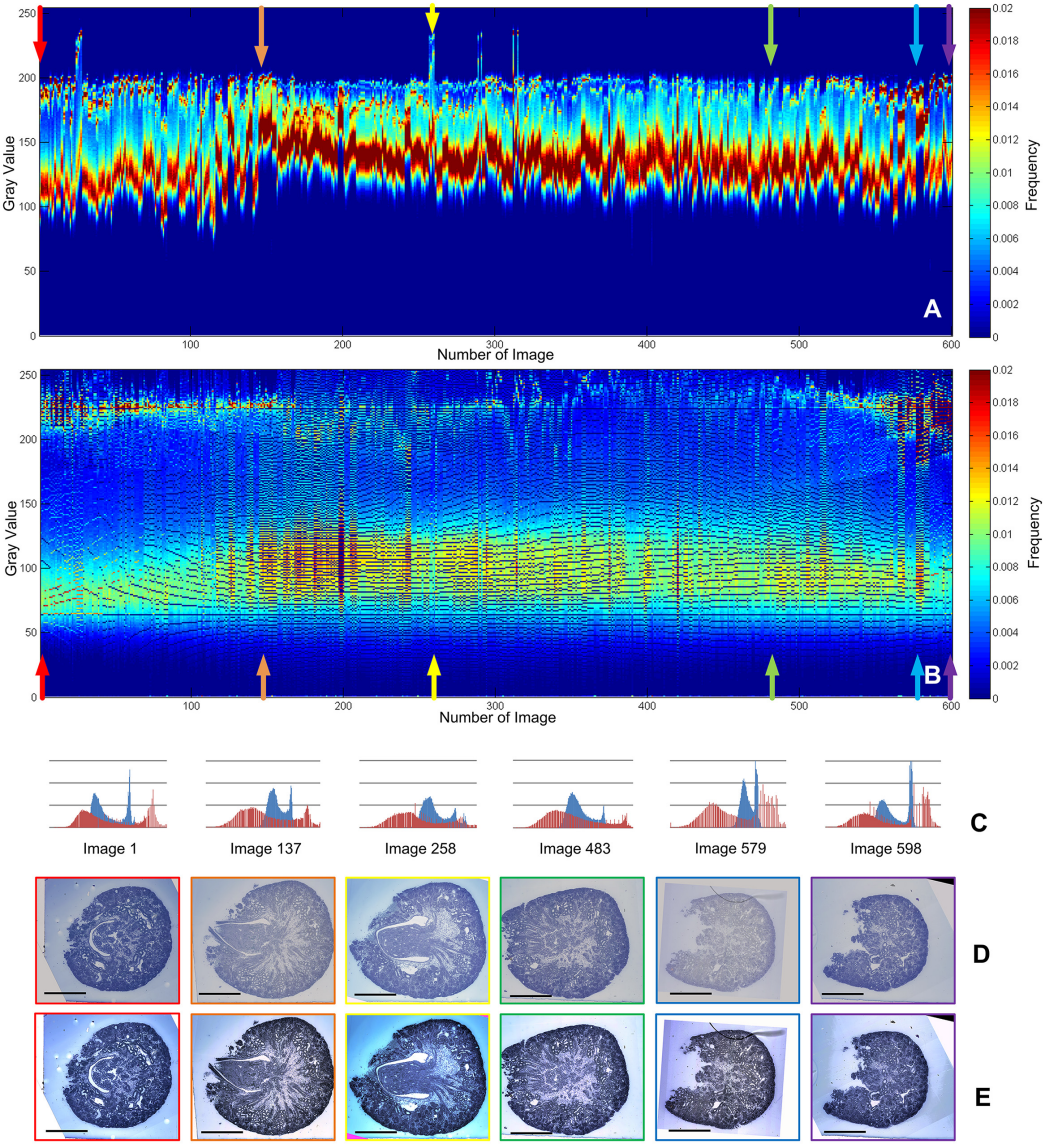
The comparison of result of the E17 kidney image stack before and after processing by SHFA. (A) Histograms of the original image stack; (B) Histograms of the image stack processed by SHFA; the abscissa represents the image number, the ordinate represents gray value (0–255), and pseudo-color represents the frequencies of gray values appearing. (C) Histograms of typical images in the stack (blue for before and red for after processing), the original and processed images are shown in (D) and (E). (Image 1: good quality, Images 137 and 483: low contrast, Image 258: over-bright, Image 579: extreme low contrast). The scale bar represents 0.5 mm for all the images in D and E.

Firstly, all the image histograms were calculated, and nine landmarks (L_*i*1_—L_*i*9_) were selected in the dynamic range of each histogram, as plotted in solid lines, Fig 2. Next, a gray value of 64 was detected as the desired gray value of landmark L'_*i*1_, and a gray value of 228 was detected as L'_*i*9_. Then, a fitting algorithm was used to calculate the desired landmarks (L'_*i*2_—L'_*i*8_), as plotted in dashed lines, Fig 2. Afterwards, the desired histograms were calculated according to the relationship between the original and desired landmarks, Fig 3B. Finally, the gray values were transformed.

In the original image stack, the gray values were changing dramatically and irregularly from image to image, and the dynamic ranges were located in a narrow band in the middle-to-upper part of each histogram (Fig 3A), reflecting inhomogeneous brightness and low contrast. Although the gray value distribution was changing dramatically and irregularly (Fig 3A), the proportion of two adjacent landmarks in the dynamic range was identical between the neighboring images. After processing, the histograms of the stack became regular, the dynamic ranges covered a designated area (Fig 3B), reflecting a homogeneous brightness and enhanced contrast. Representative images with different staining and contents were selected to show the results of the algorithm: Image 1 was of good quality, whereas Images 137 and 483 had low contrast, Image 258 was insufficiently stained and over-bright, while Image 579 had extremely low contrast and its tissue content was different from the other images. These original and processed images are shown in Fig 3D and 3E. Their histograms are shown in Fig 3C (blue for original images, red for processed images). The histograms of the processed images have kept all the features of the original histograms, indicating the detailed gray values have been preserved.

**Fig 4:**
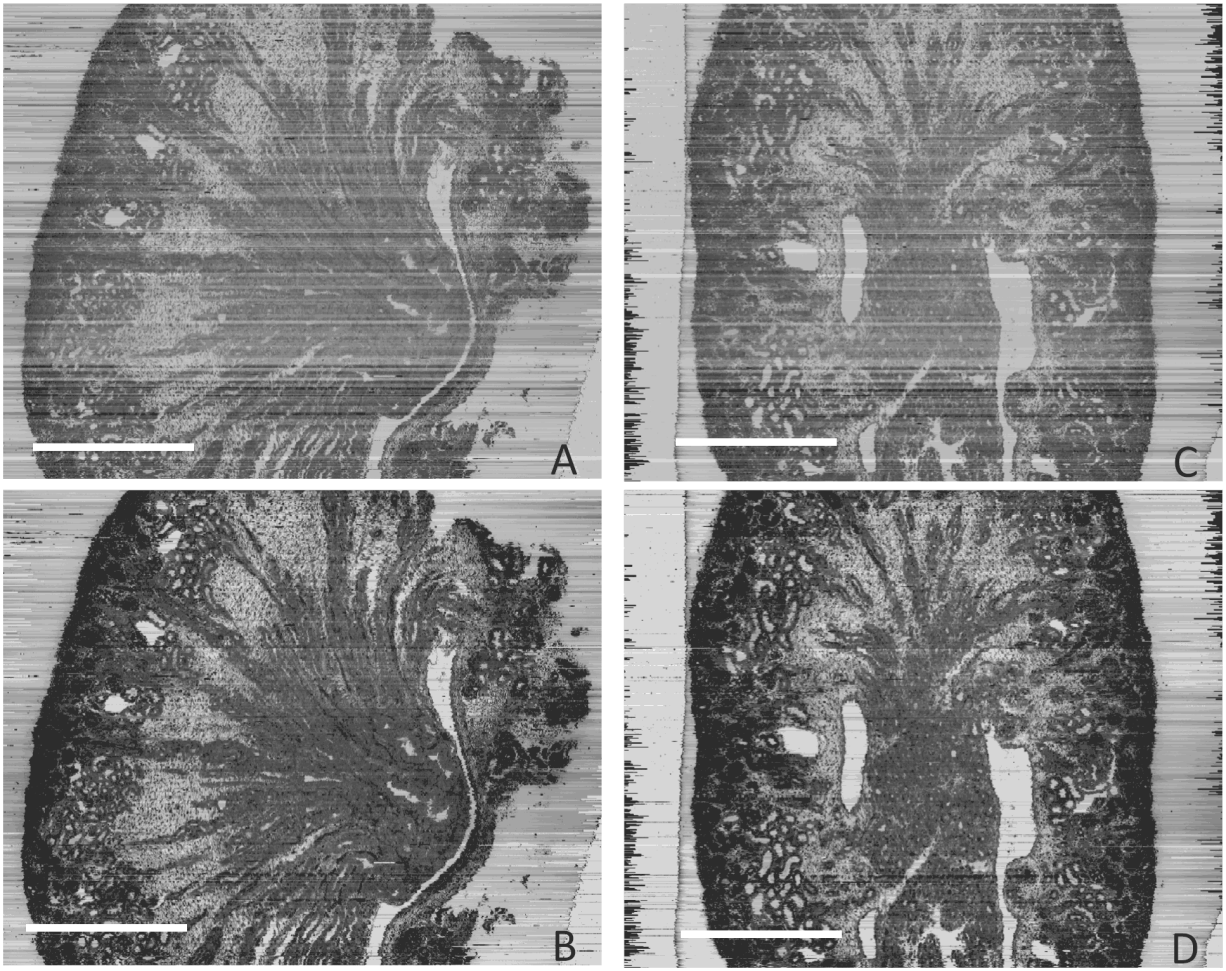
The resliced sections of three-dimensional reconstruction using the image stack of E17 kidney. (A, C) Resliced images based on the original images; (B, D) Resliced images based on the images processed by SHFA, (A, B front view; C, D lateral view). The scale bar in A represents 0.5 mm, and for B, C, and D.

The result was also evaluated by visual inspection of resliced images from the three-dimensional reconstruction based on the image stack, Fig 4. The stripes in the SHFA-processed images (Fig 4B and 4D) were less pronounced than before SHFA processing (Fig 4A and 4C). These stripes results from unequal brightness of the sections, clearly disturb the histology study and data analysis. In addition, the contrast was enhanced, thus more subtle details could be recognized in the SHFA-processed resliced images.

### Sample 2: Image Stack of P7 Kidney

**Fig 5:**
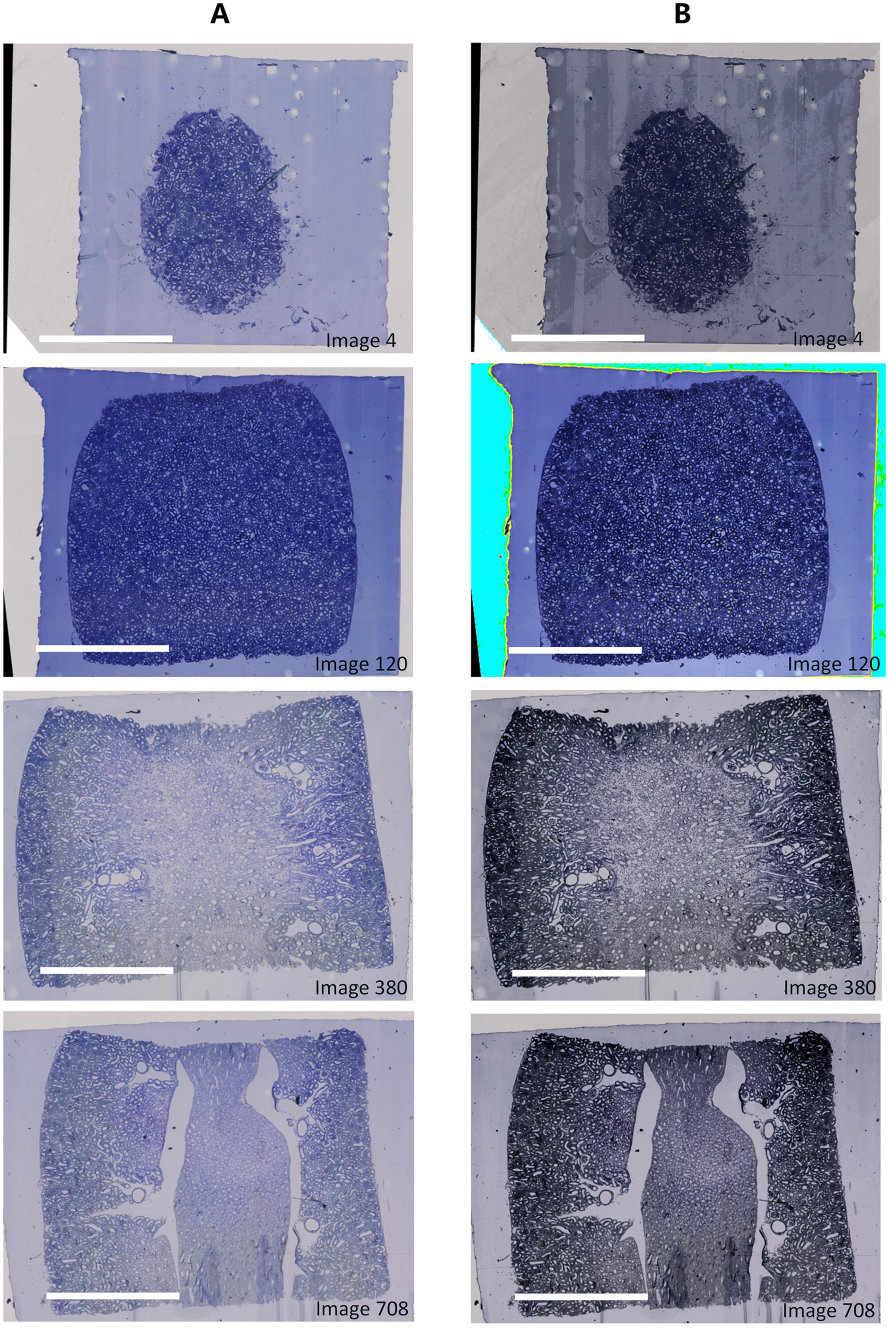
Results of the typical images from the stack of P7 kidney processed by SHFA. (A) Original images; (B) Processed images. The scale bar represents 1 mm for all the images.

The proposed algorithm was also tested with an image stack from the P7 kidney, where the tissue structures changed more dramatically in size and shape from the very first image to the middle one, and to the very last one due to the distinctive morphogenesis of an external cortex and an internal medulla. In this kidney, more tubules with thicker walls were located in the cortex than in the medulla, so that more dark stained areas were observed in cortex than in medulla. Furthermore, the convex shape of the tissue block changed from the first to the last section. In this case, SHFA proved a robust tool with a big capability for compensating uneven brightness and low contrast in a large number of serial images, Fig 5.

**Fig 6:**
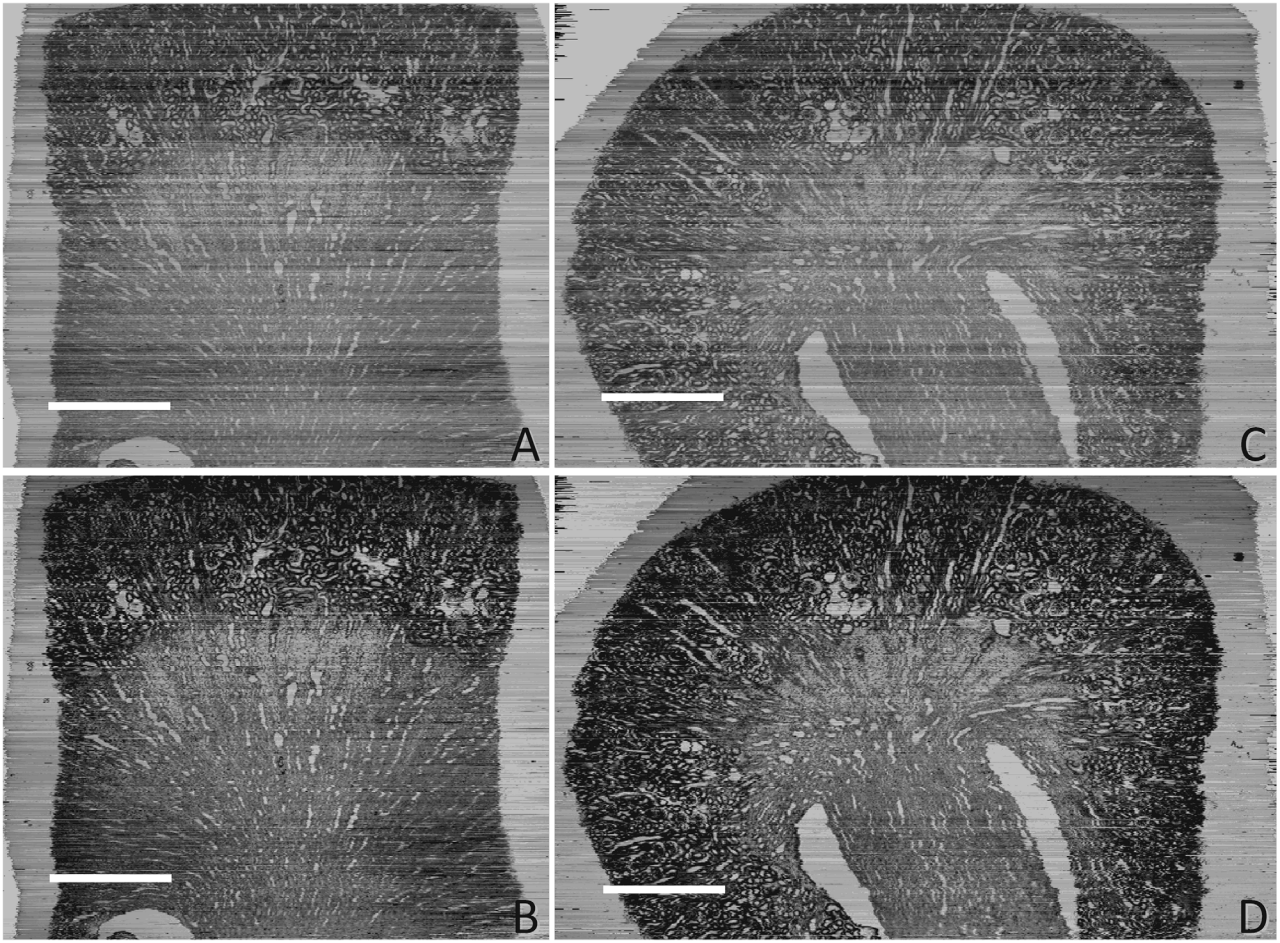
The resliced sections of three-dimensional reconstruction using the image stack of P7 kidney. (A, C) Resliced images based on the original images; (B, D) Resliced images based on the images processed by SHFA, (A, B front view; C, D lateral view). The scale bar in A represents 0.5 mm, and for B, C, and D.

The stripes in the resliced images (Fig 6B and 6D) were less pronounced than before SHFA processing (Fig 6A and 6C). The stripes in the resliced images (Fig 6B and 6D) were reduced by SHFA (Fig 6A and 6C). In addition, the SHFA-processed resliced images (Fig 6B and 6D) also revealed more detailed information.

## Discussion

Brightness compensation and contrast enhancement is a critical procedure in serial sections analysis. The present study provides a reference-free method that may better adjust a large number of images from histological serial sections. In this section, we explain its advantages in comparison with the existing methods by quantitative and qualitative analysis.

Quantitative analysis was carried out using two indicators: Kullback-Leibler Divergence (KLD) and Contrast per Pixel (CPP).

Kullback-Leibler Divergence is a non-symmetric measure of the difference between two probability distributions P and Q in probability theory and information theory. KLD is often intuited as a metric or distance, the KLD of Q from P is defined to be

$$\text{KLD(P//Q)}=\sum_{i}P\left(i\right)\ln\frac{P\left(i\right)}{Q\left(i\right)}$$

Here, *P*(*i*) represents the histogram distribution of the original image, and *Q*(*i*) represents the histogram distribution of the processed image. In the evaluation of KLD, the histogram shift in different methods can be measured.

**Fig 7:**
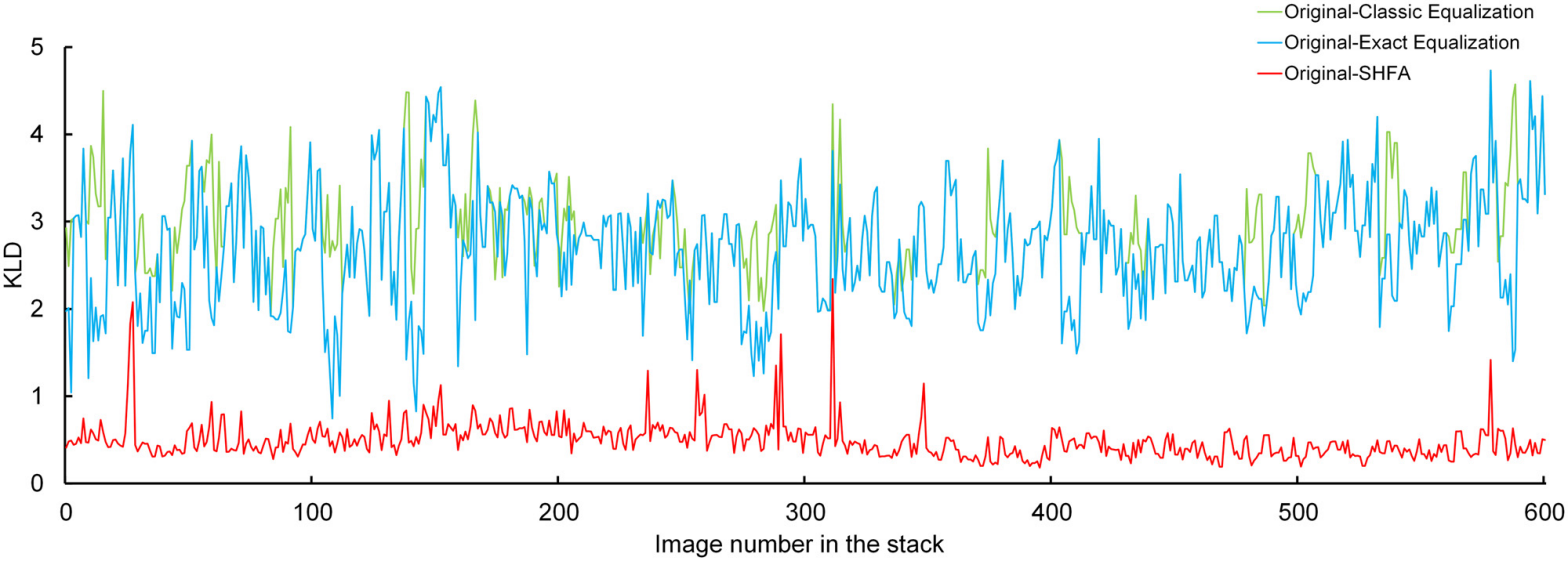
Kullback-Leibler Divergence between the histograms of original and processed images in the stack of E17 kidney.

In the image stack of E17 kidney, the KLD values are plotted in Fig 7 and the averaged values of KLD are presented in Table 1. We have found the following features: 1) the KLD varies dramatically and irregularly within each of the three groups, likely because the gray values change dramatically within the original image stack. 2) The KLD of the original-SHFA group is lowest compared with the corresponding groups by the other two methods, which could mean that the original distribution of the gray values are preserved to the largest extent. Noticeably, the KLD values for the images from 501 to 600 are highest within the groups in the cases of Classic Equalization and Exact Equalization, but not in the SHFA. Conclusively, SHFA could provide more exact desired histograms to conduct gray value transformation compared to the other two methods, when dealing with a large image stack.

**Table 1:** Averaged KLD in the image stack of E17 kidney by different methods.

Image Number	1–100	101–200	201–300	301–400	401–500	501–600
Original-Classic Equalization	3.024	2.988	2.800	2.704	2.774	3.277
Original-Exact Equalization	2.536	2.826	2.604	2.589	2.532	2.968
Original-SHFA	0.505	0.602	0.596	0.415	0.405	0.405

Original-Classic Equalization represents the KLD between the original images and the images processed by Classic Equalization, so are the Original-Exact Equalization and Original- SHFA.

**Fig 8:**
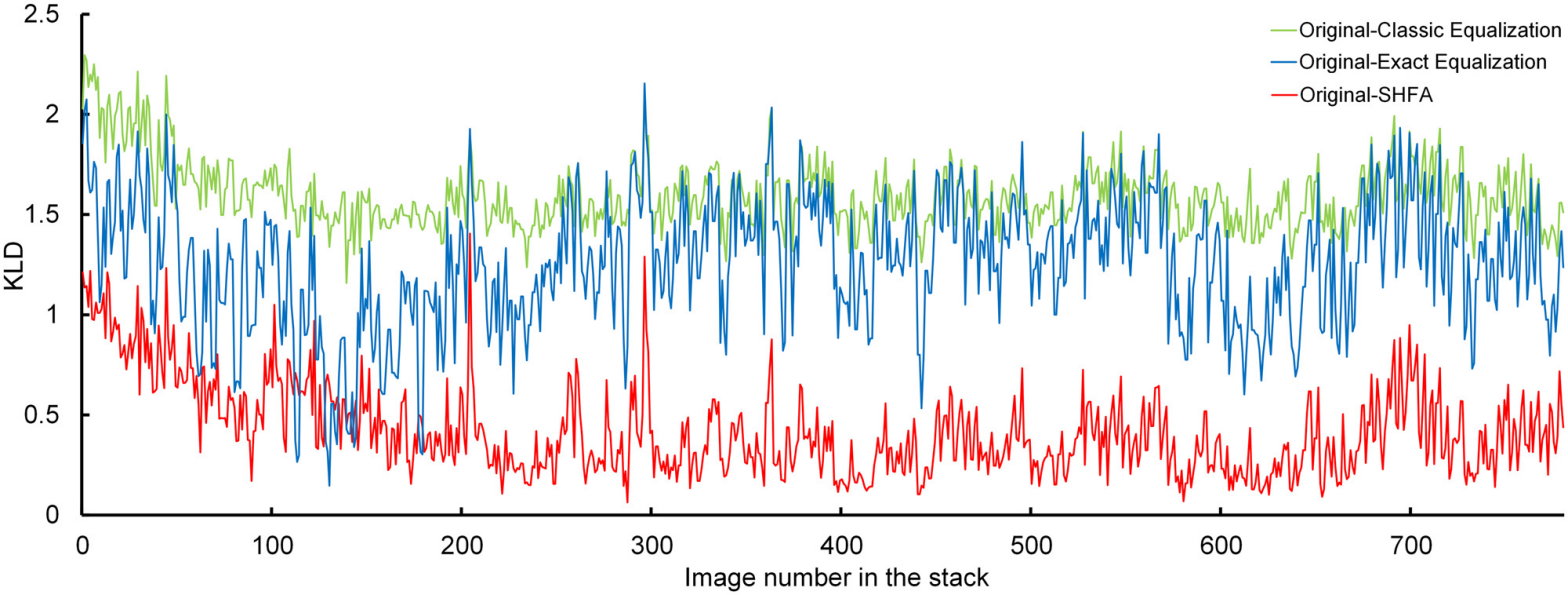
Kullback-Leibler Divergence between the histograms of original and processed images in the stack of P7 kidney.

In the image stack of P7 kidney, the KLD is plotted in Fig 8, and the averaged KDL values are presented in Table 2. Conclusions similar to those of the previous case could also be drawn in this case.

**Table 2:** Averaged KLD in the image stack of P7 kidney by different methods.

Image Number	1–100	101–200	201–300	301–400	401–500	501–600	601–700	701–780
Original-Classic Equalization	1.809	1.515	1.556	1.598	1.553	1.601	1.552	1.589
Original-Exact Equalization	1.313	0.901	1.261	1.391	1.392	1.356	1.189	1.288
Original-SHFA	0.744	0.492	0.374	0.335	0.317	0.348	0.333	0.408

Original-Classic Equalization represents the KLD between the original images and the images processed by Classic Equalization, so are the Original-Exact Equalization and Original- SHFA.

Contrast Per Pixel (CPP) [20] is an estimation of the average intensity difference between a pixel and its adjacent pixel. The CPP of an image is defined to be

$$\text{CPP}=\frac{\sum_{i=0}^{M}\sum_{j=0}^{N}\left(\sum_{\left(m,n\right)\in R_{3}^{\left(i,j\right)}}\left|f\left(i,j\right)-f\left(m,n\right)\right|\right)}{MN}$$

Here, *f*(*i*,*j*) is the gray value of pixel (*i*,*j*), and *f*(*m*,*n*) is the gray value of neighboring pixel (*i*,*j*) in the 3×3 window.

In the image stack of the E17 kidney, the CPP values are plotted in Fig 9, and the averaged CPP values are presented in Table 3. The CPP values of all the three processed image stacks have increased compared to those in the original stack, which means that the contrasts have been enhanced after processing by all the methods. The CPP of the image stack processed by Classic Equalization is highest, which implies an over-enhanced contrast. This is caused by over-stretching the histogram, and results in losing the detailed information, especially in the dark or bright tissue contents. The images processed by Exact Equalization get higher CPP values (Images 101–400), suggesting that the original image histograms are similar to the reference image histogram, but get lower CPP values when the image histograms are very different from the reference image histogram (Images 1–100 and 501–600). This situation happens when the original histograms are forced to be equivalent to the reference histogram when they are not similar. This causes an imprecise gray value transformation, losing some gray levels in the images differing from the reference image. The CPP values of the images processed by SHFA are modest, which could also be proven by images comparison, indicating that the contrast is properly improved for images both well-stained and poorly-stained, both similar and dissimilar to the reference image.

**Fig 9:**
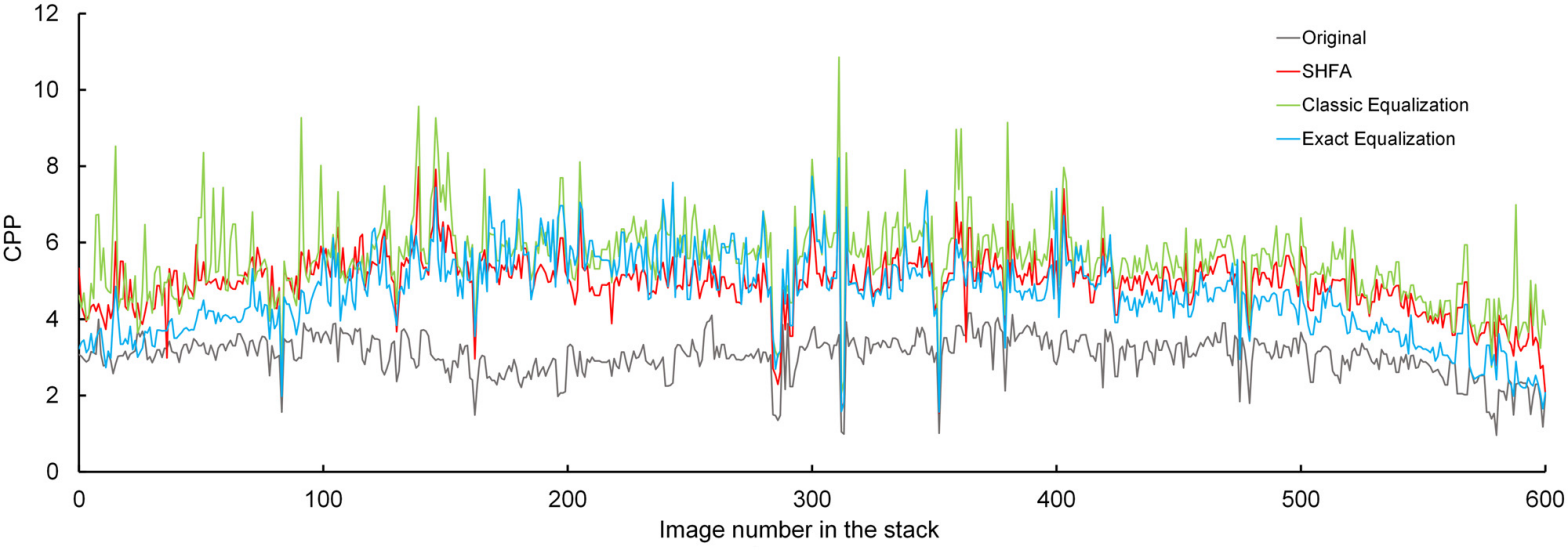
Contrast Per Pixel of the original and processed images in the stack of E17 kidney.

**Table 3:** Averaged CPP in the image stack of E17 kidney by different methods.

Image Number	1–100	101–200	201–300	301–400	401–500	501–600
Original	3.212	2.950	2.952	3.361	3.221	2.581
Classic Equalization	5.218	6.044	5.782	6.033	5.596	4.685
Exact Equalization	3.902	5.462	5.464	5.041	4.693	3.338
SHFA	4.807	5.480	4.860	5.204	5.090	4.187

**Fig 10:**
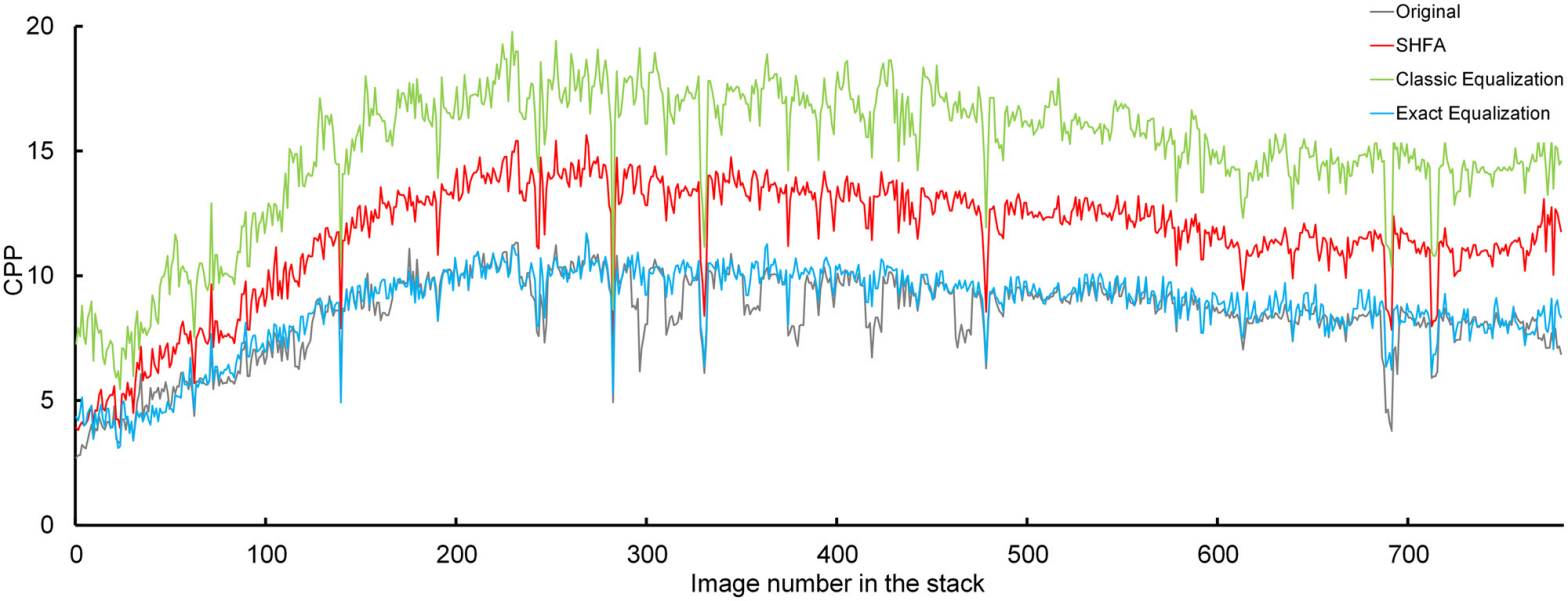
Contrast Per Pixel of the original and processed stack of P7 kidney.

The quality of the image staining in stack in P7 kidney is generally better than in E17 kidney, thus the averaged CPP values are higher, as shown in Fig 10 and Table 4. The CPP values of the image stack processed by Exact Equalization are very similar to those of the original image stack, which could be interpreted as the contrast of the detected reference image is similar to that of the other original images. This may indicate that the contrast is not improved as much in this image stack as in the E17 kidney. In the image stack processed by SHFA, the CPP values are increased but are lower in the other two processing groups, indicating that the contrast is improved at a proper level and the gray value levels of the images are better preserved.

**Table 4:** Averaged CPP in the image stack of P7 kidney by different methods.

Image Number	1–100	101–200	201–300	301–400	401–500	501–600	601–700	701–780
Original	5.141	8.746	9.943	9.363	9.094	9.063	8.060	7.869
Classic Equalization	9.248	15.49	17.38	16.97	16.66	15.83	14.37	14.17
Exact Equalization	5.282	8.957	10.14	10.01	9.601	9.252	8.500	8.125
SHFA	6.678	11.87	13.84	13.32	12.81	12.61	11.52	11.08

**Fig 11:**
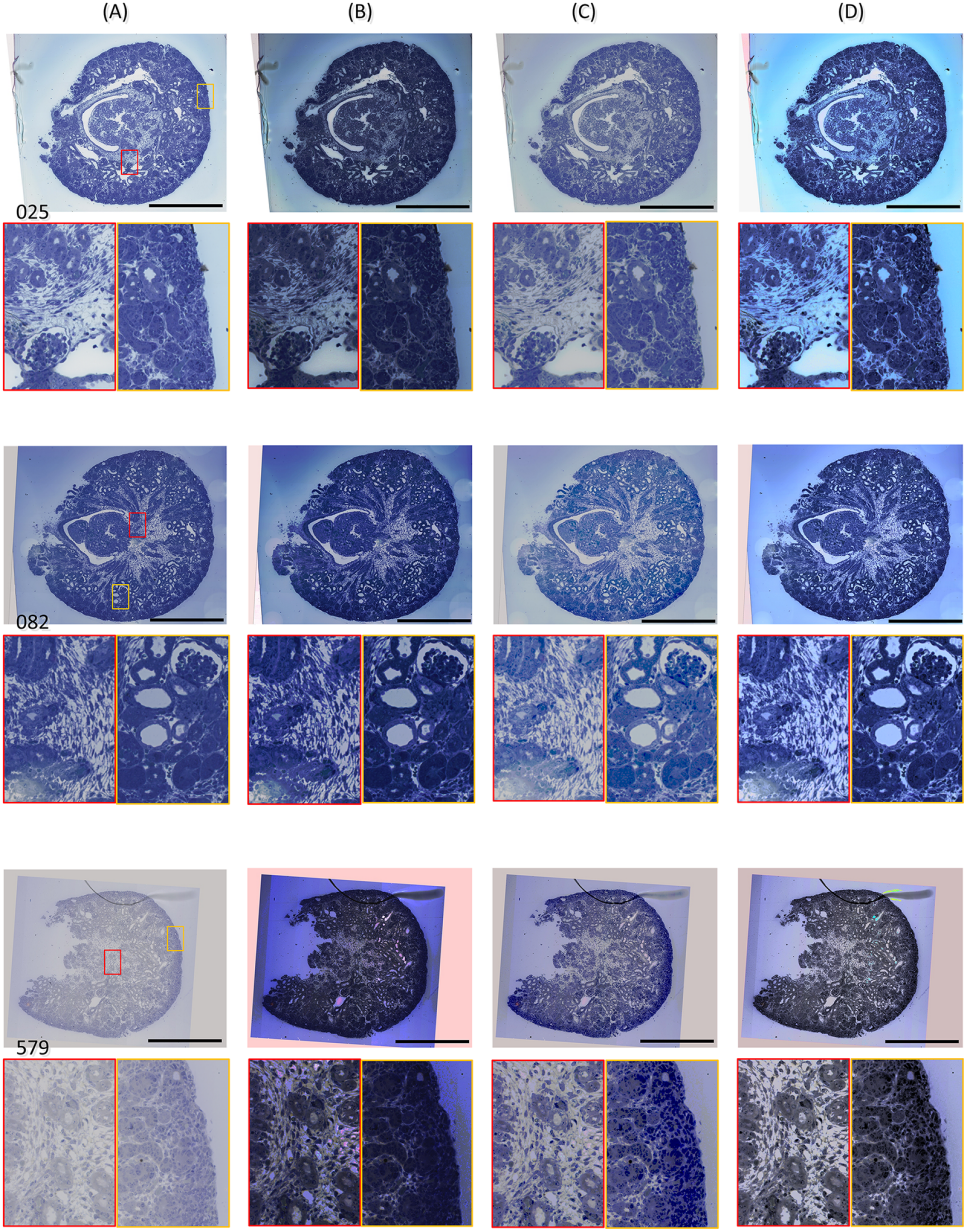
Comparison between SHFA and the existing algorithms using the image stack of E17 kidney. (A) Original images; (B) Images processed by Classic Equalization; (C) Images processed by Exact Equalization (D) Images processed by SHFA. Typical bright/black parts are magnified in the colored box. The scale bar represents 0.5 mm for all the images.

Qualitative analysis is performed by visual comparison. The global and local quality of the images in the stack is checked meticulously. Representative images in the stack of E17 kidney are illustrated in Fig 11, representing the images with good quality, deep-stained, and extreme low contrast, respectively.

By Classic Equalization, the global contrast of processed images is most enhanced among all the groups; however some gray value levels of the tissue structures are lost, especially in the darker or brighter area (Fig 11B, Images 25 and 579). The reason is that Classic Equalization focuses on the contrast of the whole image rather than a part of the image. This is a weakness in biological micrographs with a broad background and small structure area. When dealing with such images by Classic Equalization, the contrast of the background is over-enhanced, while the contrast of the tissue structures is less enhanced. Because the histogram of the background is often over-stretched, the tissue is compressed.

By Exact Equalization, a reference image is first selected visually or automatically [15], and then according to its histogram, those of the other images are adjusted, so that the contrast of the images stack is appropriately enhanced. So, the reference image with the best quality is the key of the algorithm. This algorithm performs well for the images (e.g. Fig 11C, Image 82) similar to the reference. But when a stack contains a large number of images from biological sections, or the contents in the images change dramatically (e.g. Fig 11C, Image 579), Exact Equalization would work less effectively since a single reference image could hardly represent all images in the two cases.

The presented SHFA, suitable for a larger number of images in the stack, could show the changing tendency of the original histograms, then calculates the desired histograms for gray value transformation of original images in the stack. The tendency contains most useful information from tissue structures in the images, therefore a few poorly-stained images will not affect the desired histograms. The histograms of images with abnormal brightness or low contrast (e.g. Fig 11D, Image 579) could be fitted as well.

When images processed only by SHFA are compared with those processed by SHFA and then subjected to further contrast enhancement, the latter are often preferred by observers, although both pairs of images are acceptable. This benefit of further contrast enhancement is most apparent when the original images are of good quality (e.g. Fig 11D, Image 82).

The image stack of the P7 kidney is taken as another example where the tissue content dramatically changes. In this case, the number of landmarks in the histograms, and the fitting algorithm can be adjusted according to the complexity of the changing content. In addition, since the thickness of the biological sections is at a micron level, the tissue structures rarely vary dramatically from one section to the next. According to our experience, cubic or quartic polynomial fitting algorithms are usually suitable for a large number of images from biological serial sections.

In conclusion, SHFA provides a suitable reference-free method for brightness compensation and contrast enhancement of serial section images, where a reference image or function can hardly be found, compared with the existing methods.
